# Angiosarcoma of the nasal cavity: a case report

**DOI:** 10.1186/1757-1626-2-104

**Published:** 2009-01-29

**Authors:** José Luis Treviño-González, Ramiro Santos-Lartigue, Baltazar González-Andrade, Vicente J Villagomez-Ortiz, Mario Villegas, Erving Mario Venegas-García

**Affiliations:** 1 Otolaryngology Head and Neck Surgery Service, Hospital Universitario "Dr. José Eleuterio González", Universidad Autonoma de Nuevo Leon, Monterrey, Nuevo León, México

## Abstract

Angiosarcomas are malignant neoplasias of rapid growth that develop from endothelial cells. They represent 2% of all sarcomas and only 1–4% are located in the aerodigestive tract. Since 1977, only 16 cases have been reported.

We present a 33-year-old male with spontaneous epistaxis that was refractory to cauterization. During physical examination, a smooth purplish tumor of 1.5 cm × 1.5 cm was identified. A CT scan showed a nonenhanced tumor in the left nostril on the uncinate process. A biopsy revealed an intermediate-grade angiosarcoma. Surgical removal followed by radiation therapy was performed with good result. Aerodigestive angiosarcomas have a better prognosis than angiosarcomas of other locations due to better cell differentiation and the presence of early symptoms. Recurrence can occur because of tumor tissue left during resection. Our patient continues tumor free after three years.

## Background

Angiosarcomas are malignant neoplasias of rapid growth that originate from endothelial cells [1,2]. They represent 2% of all sarcomas, and of these, only 1–4% are localized in the upper aerodigestive mucosa [3,4].

They appear during middle age and prognosis depends on location, size and degree of tissue invasion.

We present a case of angiosarcoma of the nasal cavity

## Case report

**Figure 1:**
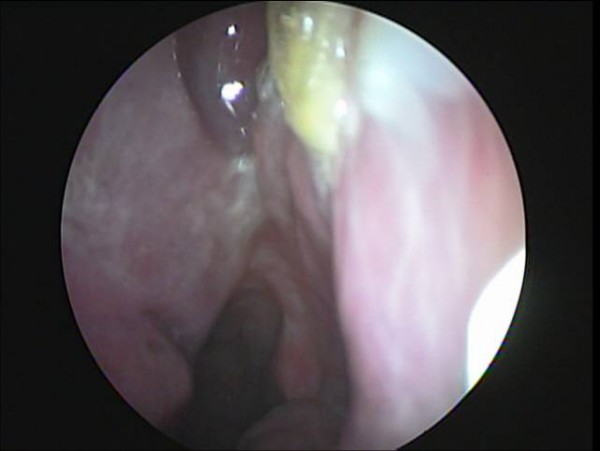
**Nasal endoscopy that shows a tumor in the left nasal wall**.

A 33-year-old male came to our clinic with a 5 month history of recurrent epistaxis of the left nostril described as nasal dripping, previously treated in another center on four occasions by cauterization of the left nasal mucosa with persistence of bleeding. During examination of the nose, we identified a smooth purplish tumor 1.5 cm × 1.5 cm attached to the lateral nasal wall between the inferior and middle turbinates (Fig 1).

**Figure 2:**
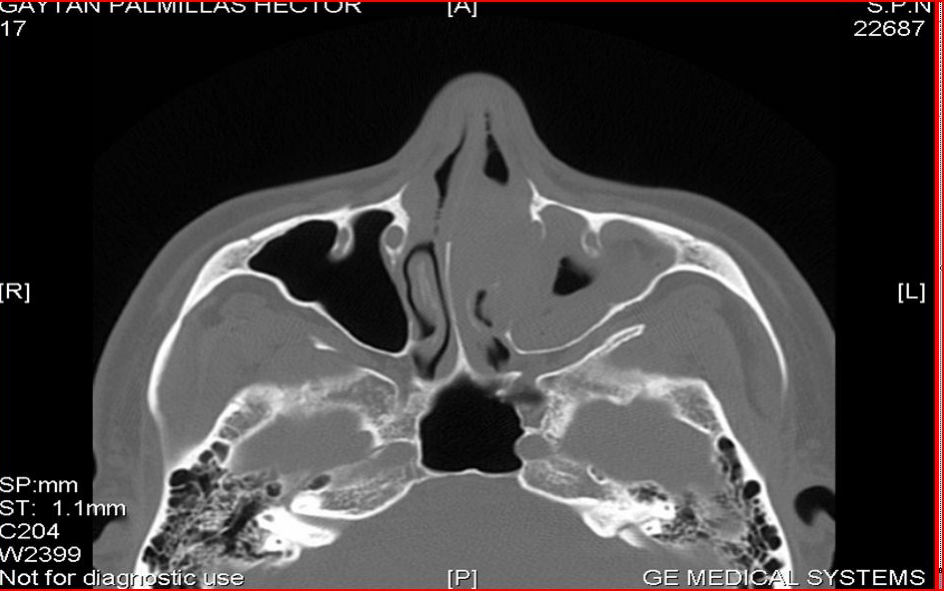
**CT Scan, of paranasal sinus, that shows the tumor in the left nasal cavity**.

A CT Scan showed a nonenhancing 3 cm. tumor in the left nasal cavity located between the inferior and middle turbinates at the level of the uncinate process with medial maxillary wall erosion (Fig 2).

Resection with maxillary antrostomy, ethmoidectomy, frontal and ipsilateral sphenoidotomy was performed. Free margins were reported on the surgical specimen. Thirty radiation cycles were applied until a total dose of 55 Gy was achieved.

**Figure 3:**
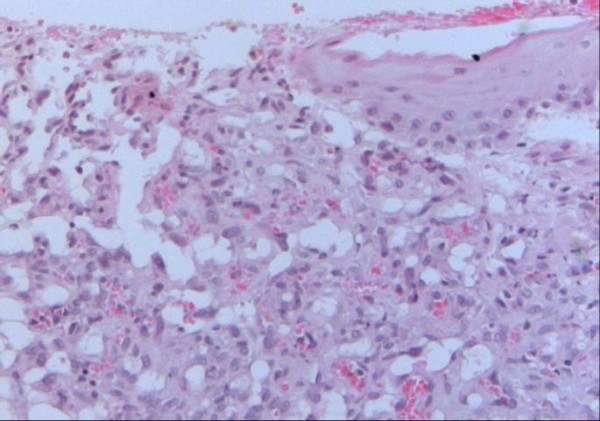
**Image that corresponds to a vascular neoplasia, composed of multiple blood vessels surrounded by endothelial cells with atypical nuclei**.

**Figure 4:**
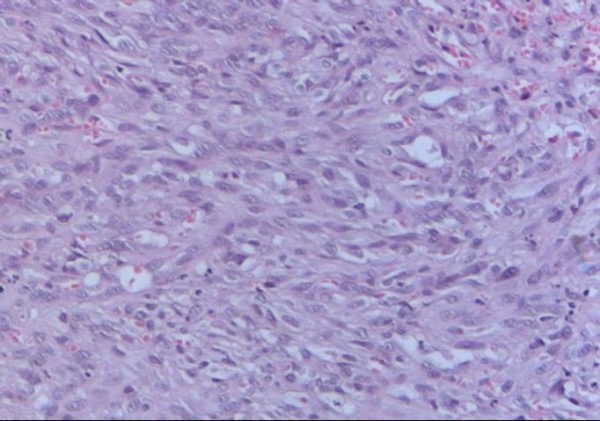
**On the solid areas of the tumor we observed elongated atypical cells with fusiform nuclei between vascular areas**.

Microscopic review of the specimen revealed a vascular neoplasia with multiple vessels comprised of endothelial cells with prominent nuclei and atypia (Fig 3). Elongated fusiform nucleus cells with atypia were also observed between the vascular areas (Fig 4). The final diagnosis was an intermediate-grade angiosarcoma.

**Figure 5:**
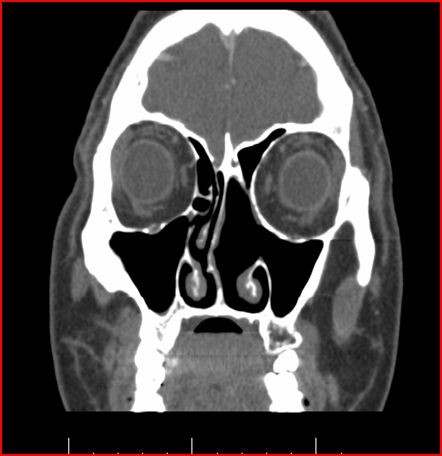
**3 years postoperative CT Scan showing nasal cavity with no presence of tumor**.

The patient continues symptom-free and with no evidence of local or distant metastasis 3 years after initial treatment (Fig 5).

## Discussion

Upper aerodigestive angiosarcoma is a very rare tumor; only 16 cases have been reported since 1977 (Table 1). Of these, six were located in the paranasal sinuses and four in the nasal cavity. Salomon [5]. from the MD Anderson Cancer Center in Houston reported that angiosarcoma corresponds to 1% of all sarcomas.

**Table 1:** Reported angiosarcoma cases, 1974–2008

Author	Case	Age	Sex	Location	Size	Time of evolution before diagnosis	Symptoms	Treatment
Pisani	1	67	F	Hypopharynx	4 cm	NR	Dysphagia and dysphonía	Total pharyngolaryngectomy + RT
Solomons	2	33	M	Maxillary sinus	NR	6 months	Epistaxis	Maxillectomy + RT
Kimura	3	8	M	Nasal cavity	NR	12 months	Epistaxis	Maxillectomy + Rhinotomy
Maheshwar	4	76	M	Oropharynx	7.5 × 4.5 cm	7 months	Throat pain	pharyngopalatectomy + RT
Mcclatchey	5	26	F	Maxillary sinus	NR	1 month	Epistaxis	RT
Williamson	6	48	M	Maxillary sinus	NR	6 months	Pain and malar edema	RT + Maxillectomy
Bankaci	7	68	M	Maxillary sinus	NR	36 months	Epistaxis, pain and diplopia	Maxillectomy + RT
Sharma	8	10	M	Maxillary sinus	NR	NR	molar pain and maxillary edema	Maxillectomy + RT
Lanigan	9	73	M	Maxilla and maxillary sinus	5 × 3 cm	2 months	Intraoral mass and hemorrhage	Hemimaxillectomy + RT
Zacharides	10	68	F	Maxilla	NR	2 months	Orbital pain	Chemotherapy
Zakzewska	11	58	M	Maxilla	NR	6 months	Hemorrhagic lesion	Maxillectomy
Kurien	12	38	M	Nasal cavity	4 × 2 cm	2 months	Nasal obstruction	RT + Maxillectomy
Oliver	13	69	F	Oral cavity	NR	3 months	Facial paralysis	Chemotherapy
Ferlitio	14	73	M	Larynx	2 cm	8 months	Dysphagia	Pharyngotomy
Ordóñez	15	52	F	Nasal cavity	5 × 4 cm	2 months	Cephalalgia and epiphora	Cranial-facial Resection + RT
Fukushima	16	55	M	Nasal cavity	NR	4 months	Epistaxis	Recombinant IL-2 + Rhinotomy + PO RT
Treviño	17	33	M	Nasal cavity	3 cm	5 months	Epistaxis	Ucinate process and lateral nasal wall resection, partial turbinectomy of the middle turbinate + RT

NR – not referred; RT – radiotherapy; PO – Postoperative

We did not find any accurate report of angiosarcoma in the nasal cavity after a review of the literature.

Nasal obstruction, malar edema and recurrent epistaxis are usually present at an early phase of the disease. The period of time for the presence of symptoms of angiosarcomas of the head and neck is short, usually one month [6]. This contrasts with angiosarcomas of the nasal cavity and paranasal sinus which is usually 7.1 months [7]. Our patient presented with a 5 month history of these symptoms.

Etiology is unknown and has been associated to certain risk factors such as chronic lymphedema, radiotherapy, vinyl chloride exposure, trauma and telangiectatic skin lesions [8,9]

The histology of aerodigestive angiosarcomas is similar to that of angiosarcomas at other locations. It is characterized by multiple vascular anastomosis, sometimes having solid necrotic or hemorrhagic areas, particularly in high-grade tumors. The neoplastic vessels present atypical endothelium with prominent hyperchromatic nuclei and can be classified in high or low grade based on their microscopic appearance [9]

Well-differentiated tumors present vascular channels with anastomosis, endothelial cells with large hyperchromatic nuclei, a low mitotic index and few pseudopapillary projections into the vessel lumen contrary to poorly-differentiated neoplasias, which are characterized by solid areas with fusiform cells [10,11]

Diagnosis of angiosarcoma is established by pathological examination of the biopsy using H&E. However, immunohistochemical staining with CD34, Ulex europaeus agglutinin I and factor VIII antigen is often required. Factor VIII-like antigen is synthesized in vascular endothelial cells and CD34 is specifically stained in vascular endothelial cells [12-14].

Radical surgery with ample margins is the treatment of choice in patients with head and neck angiosarcoma.

There is no standard treatment for nasal cavity and paranasal sinus angiosarcoma. Based on a literature review, 97% of studies reported wide resection with free margins followed by radiotherapy (4000–5000 rads) as the treatment of choice. Chemotherapy has a low impact on the disease. Kazuto Fukushima et al. reported favorable results with the use of recombinant interleukin 2, combined with surgery [9]

Free margins are the best prognostic factor for avoiding recurrence [7,8]

Angiosarcoma of the skin or soft tissue of the head and neck is associated with a 50% mortality rate within the first 25 months and a 12% survival rate at 5 years, compared to nasal cavity or paranasal sinus angiosarcoma, which have a 22% survival rate at 5 years according to grade of differentiation and early diagnosis [7]

A literature review reported a 20.8 month survival rate. Cervical metastasis occurs in 10–15% of cases and distal metastasis to bone, liver, lungs or skin occurs in 30% of patients during the first 24 months of the disease.

Radical surgery followed by radiotherapy is still the treatment of choice. Due to the lack of experience with these tumors, we believe that a better approach should be sought in order to unify criteria and provide patients with better results.

## Consent

"Written informed consent was obtained from the patient for publication of this case report and accompanying images. A copy of the written consent is available for review by the Editor-in-Chief of this journal."

## Competing interests

The authors declare that they have no competing interests.

## Authors' contributions

JT participated in the conception and design of the case, and help to draft the article. RS participated in acquisition of data and analysis and interpretation of patient data. MV participated in analysis of patient data. VV participated in critical revision and analysis of patient data. BG participated in analysis of patient data. EV drafted the article, acquisition of data and assisted in data analysis. All authors read and approved the final manuscript.
